# Optimal sequencing depth design for whole genome re-sequencing in pigs

**DOI:** 10.1186/s12859-019-3164-z

**Published:** 2019-11-08

**Authors:** Yifan Jiang, Yao Jiang, Sheng Wang, Qin Zhang, Xiangdong Ding

**Affiliations:** 1 National Engineering Laboratory for Animal Breeding, Laboratory of Animal Genetics, Breeding and Reproduction, Ministry of Agriculture, College of Animal Science and Technology, China Agricultural University, Beijing, 100193 China; 2 Shandong Provincial Key Laboratory of Animal Biotechnology and Disease Control and Prevention, College of Animal Science and Technology, Shandong Agricultural University, Taian, 271001 China

**Keywords:** Genome coverage, Sequencing depth, Pig, Whole-genome sequencing

## Abstract

**Background:**

As whole-genome sequencing is becoming a routine technique, it is important to identify a cost-effective depth of sequencing for such studies. However, the relationship between sequencing depth and biological results from the aspects of whole-genome coverage, variant discovery power and the quality of variants is unclear, especially in pigs. We sequenced the genomes of three Yorkshire boars at an approximately 20X depth on the Illumina HiSeq X Ten platform and downloaded whole-genome sequencing data for three Duroc and three Landrace pigs with an approximately 20X depth for each individual. Then, we downsampled the deep genome data by extracting twelve different proportions of 0.05, 0.1, 0.15, 0.2, 0.3, 0.4, 0.5, 0.6, 0.7, 0.8 and 0.9 paired reads from the original bam files to mimic the sequence data of the same individuals at sequencing depths of 1.09X, 2.18X, 3.26X, 4.35X, 6.53X, 8.70X, 10.88X, 13.05X, 15.22X, 17.40X, 19.57X and 21.75X to evaluate the influence of genome coverage, the variant discovery rate and genotyping accuracy as a function of sequencing depth. In addition, SNP chip data for Yorkshire pigs were used as a validation for the comparison of single-sample calling and multisample calling algorithms.

**Results:**

Our results indicated that 10X is an ideal practical depth for achieving plateau coverage and discovering accurate variants, which achieved greater than 99% genome coverage. The number of false-positive variants was increased dramatically at a depth of less than 4X, which covered 95% of the whole genome. In addition, the comparison of multi- and single-sample calling showed that multisample calling was more sensitive than single-sample calling, especially at lower depths. The number of variants discovered under multisample calling was 13-fold and 2-fold higher than that under single-sample calling at 1X and 22X, respectively. A large difference was observed when the depth was less than 4.38X. However, more false-positive variants were detected under multisample calling.

**Conclusions:**

Our research will inform important study design decisions regarding whole-genome sequencing depth. Our results will be helpful for choosing the appropriate depth to achieve the same power for studies performed under limited budgets.

## Background

Sequencing technologies have been widely used in many fields, such as human medicine [1], evolutionary chemistry [2–5], microbial ecology [6], agriculture [7] and animal breeding [8]. In sequencing, a key consideration is the sequencing depth, which is defined as the ratio of the total number of bases obtained by sequencing to the size of the genome or the average number of times each base is measured in the genome [9]. Sequencing cost is the main concern in practice, which is mainly influenced by the sequencing depth, sequencing technology and sample size. Although the sequencing cost for a particular sample has decreased significantly in recent years, it is still a great burden for large-scale applications.

Sequencing depth has a great impact not only on sequencing cost but also on the biological results of sequencing data processing, e.g., the genomic assembly completeness and accuracy of a de novo assembly [10], the number of detected genes and expression levels in RNA-Seq [11], the proportion of rare variants and SNVs detected [12], and the accuracy of SNP calling and genotyping in whole-genome sequencing [13]. Therefore, it is particularly important to investigate sequencing depth to achieve a higher accuracy at a lower cost and to identify trade-offs between sequencing data quality and quantity.

Recently, there have been many studies on the impact of sequencing depth in RNA-Seq that have aimed to find the optimal sequencing depth for either de novo transcriptome assembly through the comparison of different nonmodel animals [14] or the study of gene expression in RNA-Seq [11]. In addition, similar plant studies have been carried out to investigate the optimal transcriptome coverage in *Hevea brasiliensis* [15]. For DNA sequencing, the research on sequencing depth has mainly focused on de novo genome assembly [10, 16] and genetic association studies of complex traits [17, 18]. Additionally, several studies have explored the recommended coverage for reducing indel calling errors [19] and detecting copy number variations [20] in sequencing data. The impact of sequencing depth on single-cell sequencing has also been explored recently [21, 22]. However, most of the studies on sequencing depth conducted thus far have focused on simulated data [18, 23] or real datasets from humans [13] or pilot animals with small genomes, such as *E. coli*, *S. kudriavzevii* and *C. elegans* [10]; a limitation of simulated data is that mismapping around short indels cannot be taken into account [17], and these data cannot fully mimic the real situation in sequencing. Such investigations have seldom been conducted on animals with large genomes, such as pigs, and this research is useful not only for humans but also for livestock and other mammals.

In this study, we sequenced the whole genomes of three Yorkshire boars at a sequencing depth of approximately 20X. In addition, whole-genome sequencing data from three Landrace and three Duroc boars were downloaded from the NCBI SRA database to explore the relationship between sequencing depth and biological results from the aspects of whole-genome coverage, the variant discovery power and the quality of variants.

## Methods

### Animal ethics statement

Necessary permission was obtained from the owner of the farm for collecting the samples and using in the next study. All animal management and sample collection procedures strictly followed the protocol approved by the Institutional Animal Care and Use Ethics Committee (IACUC) at the China Agriculture University. And the IACUC of the China Agricultural University specifically approved this study (Permit Number: DK996).

### Whole-genome resequencing

Blood samples from three Yorkshire boars were collected from a breeding farm in Beijing. Genomic DNA was extracted from the blood samples by using a TIANamp Blood DNA spin kit (DP348; Tiangen, Beijing) following the manufacturer’s protocol. The quality of all DNA samples was evaluated by agarose gel electrophoresis, and accurate quantification of DNA concentrations was conducted with a Qubit 2.0 fluorometer. Whole-genome sequencing was performed using the Illumina HiSeq X Ten platform according to the manufacturer’s standard protocols and produced 150-bp paired-end reads in fastq format. Whole-genome sequencing data for three Duroc and three Landrace pigs in sra format were downloaded from the NCBI SRR database and then converted to fastq format by fastq-dump in the SRA Toolkit. All individuals that we selected were unrelated to each other.

### Sequencing quality control and NGS data processing

To avoid reads with artificial bias, quality control was conducted by using the NGS QC Toolkit [24]. First, IlluQC.pl with the default parameters was used to remove reads that contained more than 30% low-quality (quality value ≤20) bases. Second, TrimmingReads.pl was used to trim the 3′ end of fragments. Then, high-quality paired-end reads were mapped to the pig reference genome sequence (Sscrofa11.1 http://hgdownload.soe.ucsc.edu/goldenPath/susScr11/bigZips/) by the BWA (Burrows-Wheeler Aligner) [25] with the command ‘bwa men -M -R’.

### SNP calling and filtering

The Genome Analysis Toolkit (GATK) [26] (version: 3.7) was used to call SNPs, following GATK best practices [27], in which realignment and recalibration were included. SNP calling and genotyping were performed by UnifiedGenotyper in GATK. Only SNPs on autosomes were used for the following analysis. Before SNP calling, Picard SortSam and Picard MarkDuplicates (http://broadinstitute.github.io/picard/) were used to sort and mark potential PCR duplicates separately. After SNP calling, hard filters were used to remove potential false-positive SNPs and InDels. For SNPs, the following criteria were used for filtering, as suggested by the GATK documentation: “QD < 2.0 || FS > 60.0 || MQ < 40.0 || HaplotypeScore > 13.0 || MQRankSum < -12.5 || ReadPosRankSum < -8.0”. For InDels, the criteria “QD < 2.0 || FS > 200.0 || ReadPosRankSum < -20.0” were used for filtering, also suggested by the GATK documentation. Both single-sample calling and multisample calling (three samples from each breed) implemented in the GATK UnifiedGenotyper were used in our analysis.

### Relationships

Principal component analysis (PCA) was performed via GCTA(a tool for Genome-wide Complex Trait Analysis) [28, 29] including all common SNPs in all individuals after filtering by the minor allele frequency (MAF ≥ 0.05) and LD pruned (--indep-pairwise 100 50 0.5). The heatmap of the genomic relationship between each individual was plotted by using a heatmap in R.

### Construction of samples with different sequencing depths

**Fig. 1:**
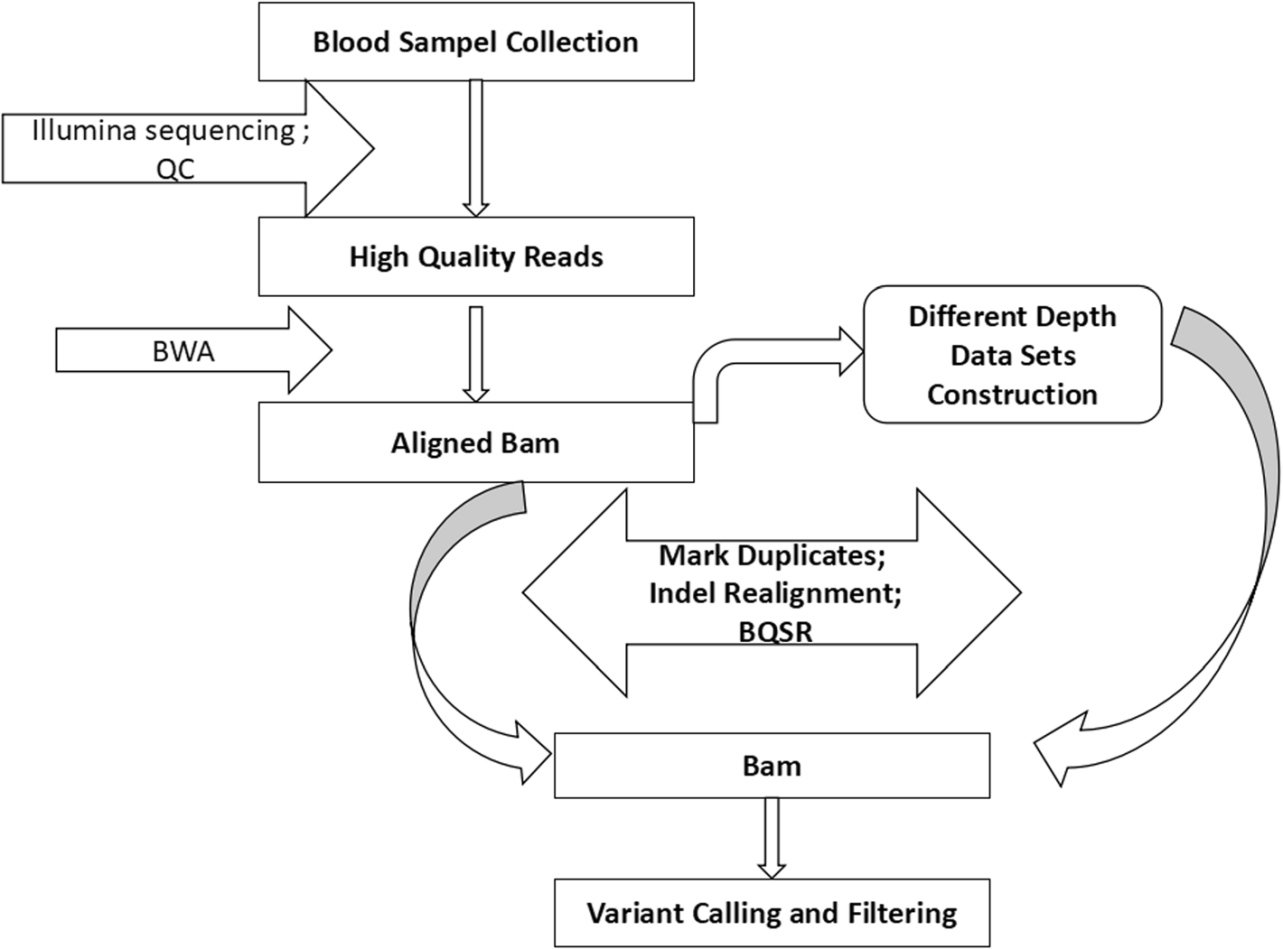
Data processing pipeline. Our pipeline was identical for each sample. Original aligned bam files were mapped with clean fastq data. Then, we extracted different proportions of paired reads randomly from the original bam files to build samples with different depths. Markduplicates, Indel realignment and Base recalibration were applied for all bam files and the same procedures were used for SNP calling and filtering

Picard DownsampleSam was used to randomly downsample a bam file to construct different lower-depth samples. In this way, mate-pair reads were either kept or both discarded. Proportions of 0.05, 0.1, 0.15, 0.2, 0.3, 0.4, 0.5, 0.6, 0.7, 0.8 and 0.9 were set in a chained strategy for the raw mapped bam file, and average depths of 1.09X, 2.18X, 3.26X, 4.35X, 6.53X, 8.70X, 10.88X, 13.05X, 15.22X, 17.40X, 19.57X and 21.75X corresponding to each proportion were produced. Together with the original greatest depth for each individual, a total of 12 gradient depths for each sample were used for further analysis. Figure 1 illustrates the workflow of next-generation sequencing (NGS) data processing.

### Variant annotation

ANNOVAR [30] was used to annotate the variants with the dbSNP database. Following Ai et al. [4], those variants that met one of the following criteria were defined as potential loss-of-function (LoF) variants: (1) a SNP or small Indel within a coding region causing a frameshift of the open reading frame (ORF); (2) a SNP or small indel at a splice site; and (3) a SNP or small indel within a coding region resulting in a stopgain or stoploss.

### Comparison of data with different depths

After variant calling and filtering, we compared data with different depths from the following aspects: (1) whole-genome coverage; (2) the number of SNPs discovered; (3) the discovery power for variants; and (4) the quality of variants evaluated by the novel rate and transition/transversion ratio.

The discovery power for each subsample is the ratio of variants in downsampled data to that in the original deep sequencing data.

The novel rate represents the proportion of variants not available in the dbSNP database to the total variants, as we defined the variants that were not included in the dbSNP database as novel variants. Here, we used the pig dbSNP database (Build ID: 150).

The transition/transversion ratio (Ti/Tv ratio) is the proportion of the variants observed as transitions (between purines, or between pyrimidines) versus transversions (between purines and pyrimidines). The Ti/Tv ratio is particularly useful for assessing the quality of single nucleotide polymorphisms inferred from sequencing data [31, 32]. A higher ratio generally indicates higher accuracy [27].

GATK DepthOfCoverage was used to compute the whole-genome coverage, and GATK VariantEval and R [33] were used to extract summary statistics from the results and for further analysis.

### BeadChip validation

To further evaluate the accuracy of SNP calling, all samples of Yorkshire boars were genotyped with the PorcineSNP80K BeadChip (Illumina, San Diego, CA, USA), including 68,528 SNPs covering the whole genome. Genotype quality control was carried out with PLINK1.9 [34], in which SNPs with call rates less than 95% and nonchromosomal SNPs were excluded. The common sites between each subdepth and beadchip dataset were counted, which were defined as common sites on the beadchip. The discordance rate for each sample and genotype caller was calculated for single-sample calling, which was defined as the fraction of the number of common sites for the sample between the PorcineSNP80K BeadChip and the whole-genome sequencing data processed by the genotype caller according to the number of genotypes that mismatched the chip genotype. For comparison between single-sample calling and multisample calling, the discordance rate for multisample calling was calculated, defined as the proportion of the number of genotype discordance sites to the common sites of the sample between the PorcineSNP80K BeadChip and whole-genome sequencing SNPs containing at least one nonreference allele.

## Results

### Summary of whole-genome sequencing and subsampling

**Fig. 2:**
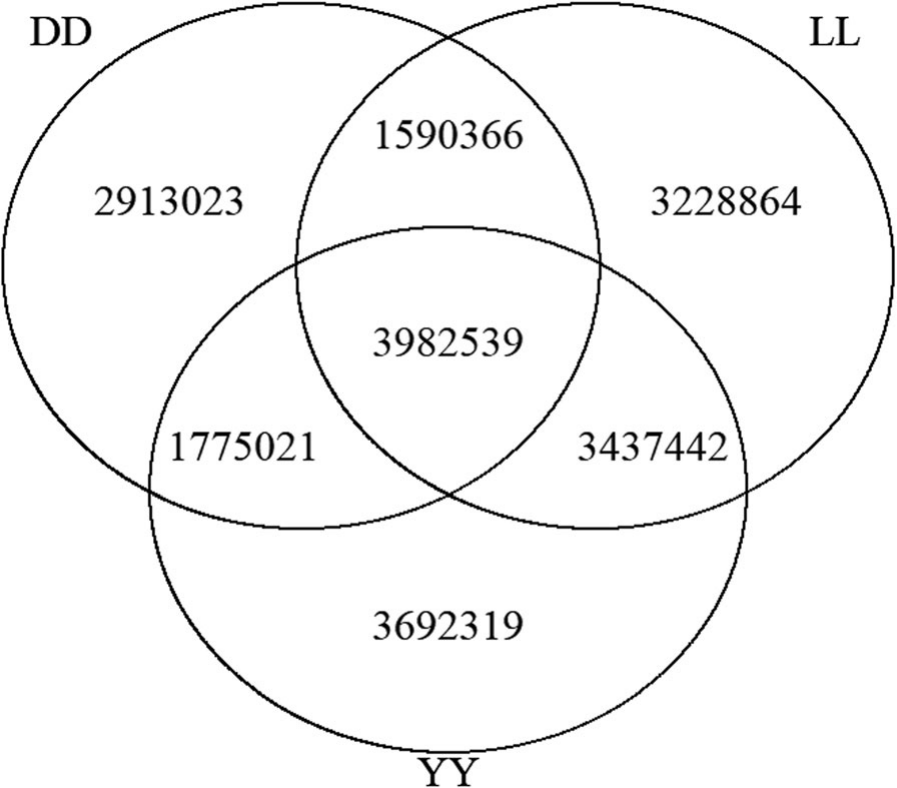
The Venn diagram shows the variants discovered in each breed. The Venn diagram shows the overlap of the variants discovered in each breed. A total of 10,260,949, 12,239,211 and 12,887,321 SNPs were discovered in Duroc, Landrace and Yorkshire, respectively. A total of 3,982,539 common variants were discovered in all three breeds, while 5,572,905, 5,757,560 and 7,419,981 common variants were discovered between Duroc-Landrace, Duroc-Yorkshire, and Landrace-Yorkshire, respectively

A summary of the sequencing data for three Yorkshire boars, three Duroc boars and three Landrace boars is shown in Table 1. The sequence data for each individual reached a depth of greater than 20-fold and covered more than 99% of the whole genome. Furthermore, multisample calling of the three individuals of each breed was implemented to perform a comparison of the SNP differences between the Duroc, Landrace and Yorkshire pigs, as shown in Table 2. A total of 10.26, 12.24, and 12.89 million variants were discovered for the Duroc, Landrace and Yorkshire pigs, respectively. Figure 2 shows the overlap of the variants discovered in each breed. A total of 5.57, 5.76, and 7.42 million common variants were discovered between Duroc-Landrace, Duroc-Yorkshire, and Landrace-Yorkshire, respectively, and 3.98 million common variants were discovered in all three breeds. Among the three pig breeds, Duroc presented the smallest number of SNPs, which was not surprising because the *Sus scrofa* reference genome comes from the Duroc breed. In addition, as shown in Table 2, when the variants were compared to the pig dbSNP database, a total of 8.67, 10.47 and 11.21 million variants were included in the dbSNP database, accounting for 84.50, 85.53 and 86.99% of the total variants of Duroc, Landrace and Yorkshire, respectively. ANNOVA annotation revealed 68,313, 78,968 and 85,372 variants in exon regions, among which there were 28,193, 31,980 and 33,538 loss-of-function (LoF) variants in Duroc, Landrace and Yorkshire, respectively. The transition/transversion ratios for Duroc, Landrace and Yorkshire were 2.24, 2.25 and 2.34, respectively. The detailed information for each individual is shown in Table 3.

**Table 1:** Summary statistics for the whole genome sequencing of data

Sample	Depth	Coverage	Map Ratio	Breed	Sex	SRA project
S403505	24.40	99.41%	99.30%	Yorkshire	male	
S474607	22.31	99.39%	99.53%	Yorkshire	male	
S494203	21.79	99.32%	98.76%	Yorkshire	male	
SAMN05791661	22.65	99.44%	99.50%	Duroc	male	PRJNA343658
SAMN05791665	21.72	99.46%	99.57%	Duroc	male	PRJNA343658
SAMN05791663	23.90	99.57%	99.50%	Duroc	male	PRJNA343658
SAMN05791650	19.89	99.30%	99.47%	Landrace	male	PRJNA343658
SAMN05791651	22.56	99.39%	99.57%	Landrace	male	PRJNA343658
SAMN05791660	19.36	99.38%	99.73%	Landrace	male	PRJNA343658

**Table 2:** Summary statistics of variants discovered for three pig breeds, Duroc, Landrace and Yorkshire

Sample	Mean Depth	Total Variants	%variants in dbSNP	Ti/Tv Ratio	Exons	LoF
3DD-sample	22.76	10,260,949	84.50	2.24	68,313	28,193
3LL-sample	20.60	12,239,211	85.53	2.25	78,968	31,980
3YY-sample	21.88	12,887,321	86.99	2.34	85,372	33,538

*Mean Depth* the sequencing depth for each breed on average, *TotalVariants* the number of SNPs discovered for each breed by 3 sample multisample calling, *%variants in dbSNP* the number of SNPs in the dbSNP database, *Ti/Tv Ratio* the proportion of the SNPs observed as transitions (between purines or between pyrimidines) versus transversions (between purines and pyrimidines), *Exons* the number of SNPs in exons, *LoF* the number of loss-of-function variants

**Table 3:** Summary statistics of variants discovered for each individual

Breed	Sample	Total Variants	%variants in dbSNP	Ti/Tv Ratio	Exons	LoF
DD	SAMN05791661	4,361,699	83.84	2.18	28,321	11,783
DD	SAMN05791665	4,720,118	84.00	2.20	31,091	13,272
DD	SAMN05791663	4,761,804	85.38	2.26	29,916	11,992
LL	SAMN05791650	4,664,664	84.42	2.16	29,287	12,430
LL	SAMN05791651	5,255,293	85.29	2.21	33,575	13,768
LL	SAMN05791660	5,433,413	86.27	2.25	35,521	14,111
YY	S494203	5,730,270	86.99	2.31	39,094	15,304
YY	S409308	5,528,070	86.74	2.30	38,986	15,630
YY	S474607	6,047,346	86.81	2.28	38,828	15,482

*TotalVariants* the number of SNPs discovered for each individual by single-sample calling, *%variants in the dbSNP databbase* the number of SNPs in the dbSNP database, *Ti/Tv Ratio* the proportion of the SNPs observed as transitions (between purines, or between pyrimidines) to transversions (between purines and pyrimidines), *Exons* the number of SNPs on exons, *LoF* the number of loss-of-function variants

**Fig. 3:**
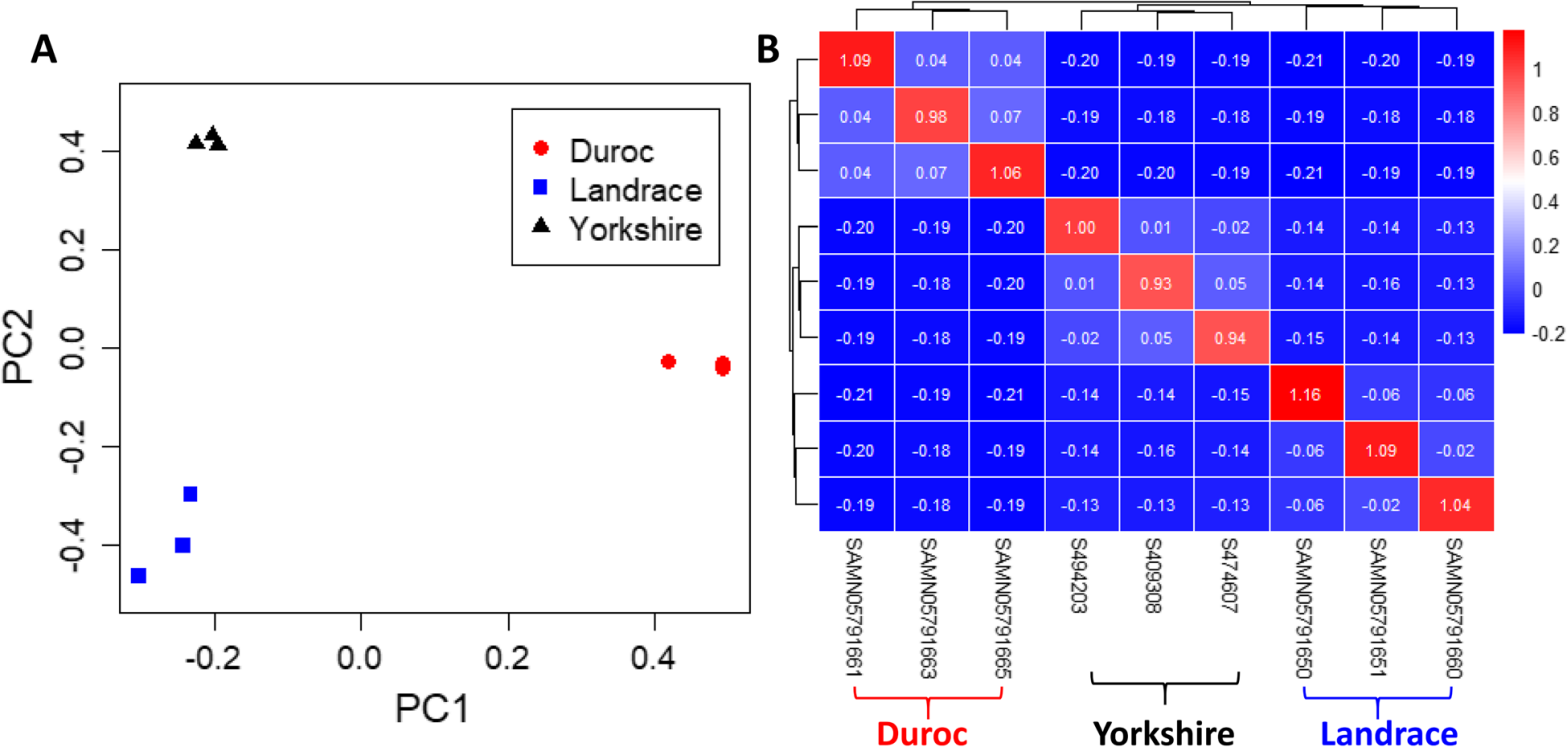
Population structure of sequenced individuals. (**a**) Principle component analysis (PCA) of individuals. Different symbols and colors indicated the different pigs by breed. PC1: first principal component; PC2: second principal component. (**b**) Heatmap of all 9 sequenced individuals using the molecular relationship matrix. The exact genomic relationship between two individuals is shown in each small lattice

The population structure was demonstrated through principal component analysis (PCA) (Fig. 3a) using 1.37 billion SNPs. Figure 3a shows that the Duroc, Yorkshire and Landrace breeds were distinctly separated from each other. The heatmap of the genomic relationships between each individual shown in Fig. 3b indicated that the three individuals of each breed were clustered together and they were unrelated individuals to each other.

### The impact of depth on the coverage

**Fig. 4:**
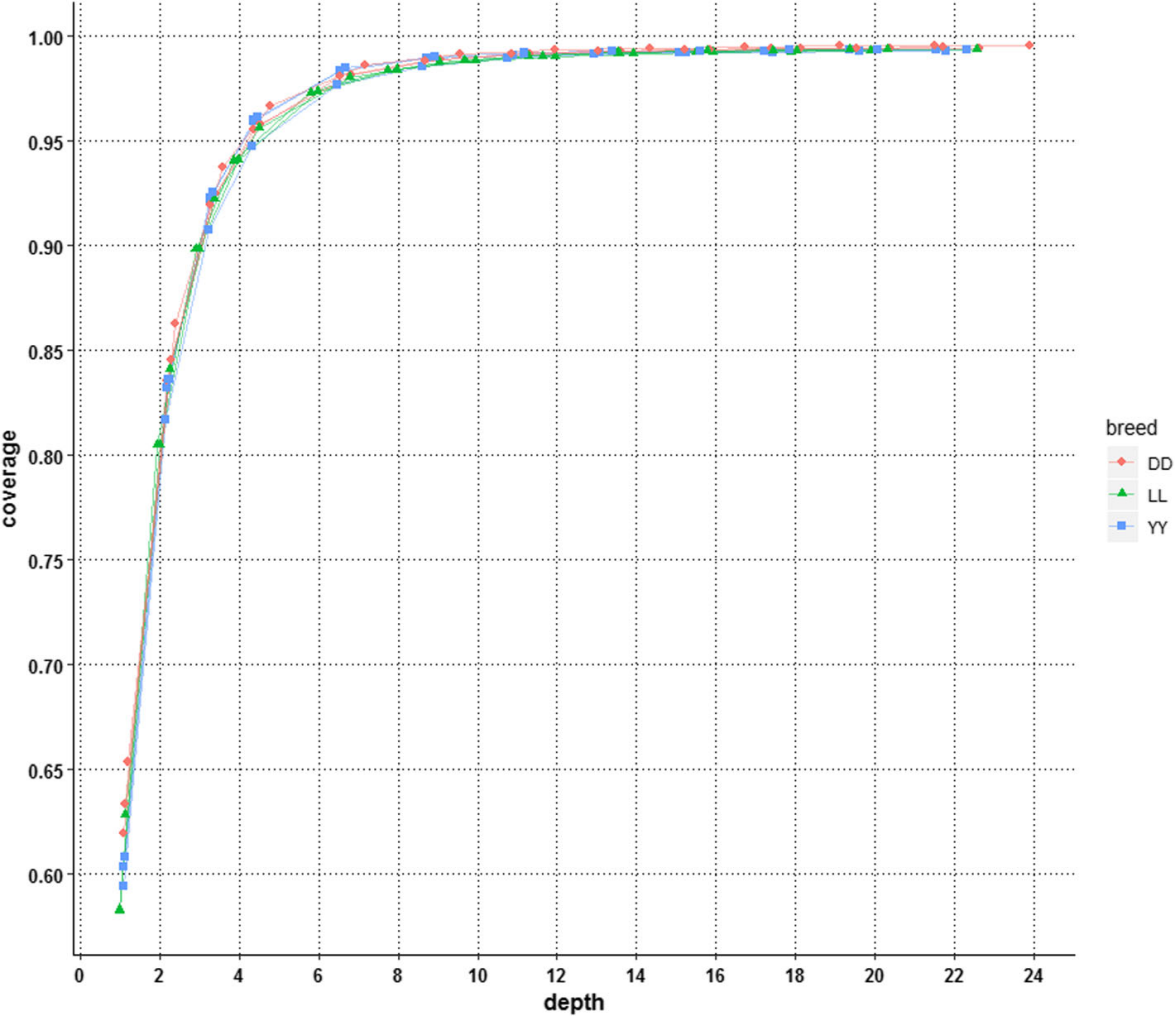
Coverage with sequencing depth for each downsampled genome. Whole-genome coverage as a function of sequencing depth for each downsampled genome

The coverage of the genome at each depth is illustrated in Fig. 4. All of the individuals from the three pig breeds presented almost the same trend, as the curves for all individuals nearly completely overlapped, and the coverage of each individual at a given depth was nearly the same. These results indicated that there was no breed or individual specificity for the relationship of coverage and sequencing depth. In general, the coverage increased with the depth, but not linearly; there was a rapid increase in coverage from 61.17 to 95.42% when the depth was increased from 1.08X to 4.35X, after which the coverage increased relatively slowly from 95.42 to 99% when the depth was increased from 5X to 10X and plateaued at a depth of 10X (at a proportion of 0.5 on average), which covered ~ 99% of the whole genome and was very close to the coverage achieved at depths of 13.05X, 15.22X, 17.40X, 19.57X and 21.75X of 99.21, 99.29, 99.34, 99.37 and 99.40% of the whole genome, respectively (Fig. 4). According to the curve presented in Fig. 4, a depth of 4.35X (at a proportion of 0.2 on average) was the inflection point, at which the coverage increased exponentially (< 4.35X) and the increase ratio then decreased slightly. At the inflection point, ~ 95.42% of the genome was covered.

### Discovery of variants and quality of variants

**Fig. 5:**
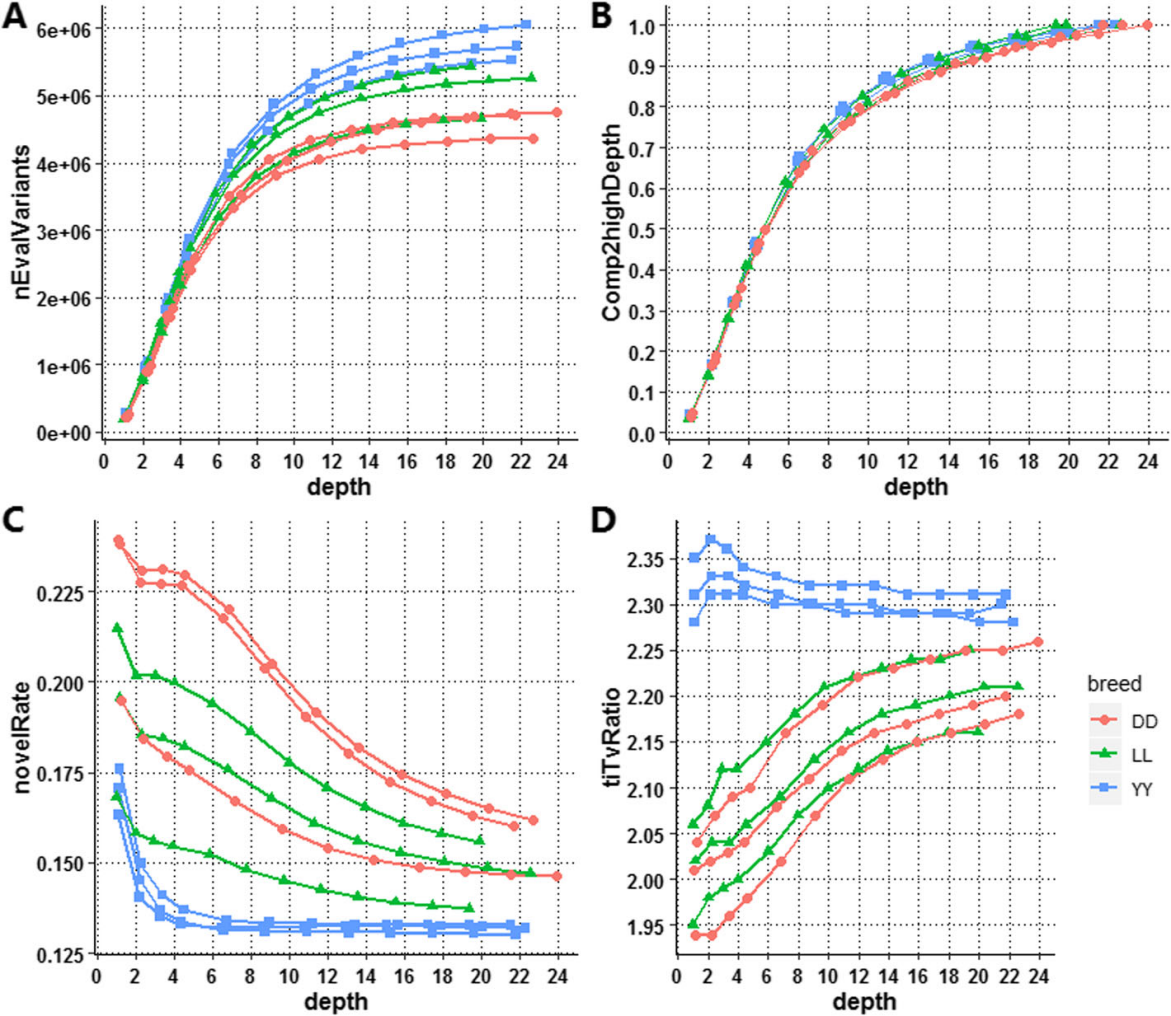
Discovery power and accuracy of SNP calling. (**a**) The number of total variants (only SNPs) discovered for each downsampled dataset. (**b**) Total variant discovery power. We refer to the variants associated with the greatest depth as true variants for each sample and calculate the discovery power for each subdepth, which is the proportion of variants discovered at each depth among the total variants at the greatest depth for each sample. (**c**) Novel rate. The proportion of variants present in the dbSNP database among the total variants. (**d**) Ti/Tv ratio. The proportion of the variants observed as transitions (between purines or between pyrimidines) to transversions (between purines and pyrimidines)

The total variants discovered at each depth for each sample are shown in Fig. 5a. The largest number of variants was discovered in Yorkshire, followed by Landrace and Duroc, similar to the results in Table 1, and the total variants for individuals within the same breed varied as well. The discovery power for all individuals (the proportion of variants in a downsampled individual to the greatest depth for the same individual) was also slightly different across breeds (Fig. 5b). The discovery power for variants exhibited a similar tendency to genome coverage, as shown in Fig. 4, which increased with depth, and a similar tendency was demonstrated across individuals. As expected, the number of variants discovered increased rapidly when the depth was less than 10X, which was the point at which the coverage plateaued, after which the rate of increase slowed. At a depth of 10X, approximately 4.62 million SNPs were detected, accounting for 84.42% of the variants obtained from the deepest genome (21.75X in this study). The variant discovery power was increased by 15.58% on average when the depth was increased from 10.88X to 21.75X, but it increased by 80.40% on average when the depth was increased from 1.09X to 10.88X, accounting for 4.02 and 84.42% of the variants, respectively. At 4.35X, which was the inflection point of the function of coverage with depth, 45.36% (2.55 million) SNPs were detected.

The accuracy of SNP calling and genotyping was evaluated based on the novel rate and the transition/transversion ratio, as illustrated in Fig. 5c and Fig. 5d. The novel rate decreased with increasing sequencing depth (Fig. 5c), showing that the greater the depth of sequencing, the greater the number of reliable variants discovered. However, a large differences were observed between Duroc, Yorkshire, and Landrace. The sharpest decrease in the novel rate occurred in Yorkshire when the depth increased from 1.10X to 4.38X, accounting for 16.99 and 13.45% of the variants in the dbSNP database, respectively, and then remaining basically unchanged, with a ratio of ~ 13.2%, when the depth was greater than 4.38X, which may indicate that more false-positive variants were discovered when the depth was less than 4.38X. However, for Duroc and Yorkshire, the decline in the novel rate was slow when the depth was increased from 1X to 22X.

The Ti/Tv ratios for each depth in all samples ranged from 1.99 to 2.34.

as shown in Fig. 5d; Yorkshires presented higher Ti/Tv ratios than Durocs and Landraces, and the Ti/Tv ratios for Durocs and Landraces were similar. In general, the variation of the Ti/Tv ratio was not large; when the sequencing depth was increased from ~1X to ~21X, the ranges of the Ti/Tv ratio were 2.00~2.21, 2.01~2.21, and 2.31~2.30 for Duroc, Yorkshire and Landrace, respectively. Only the Ti/Tv ratios for Duroc and Landrace increased with depth, indicating higher accuracy of SNP calling. However, the Ti/Tv ratio basically remained unchanged for Yorkshire.

### BeadChip validation

**Fig. 6:**
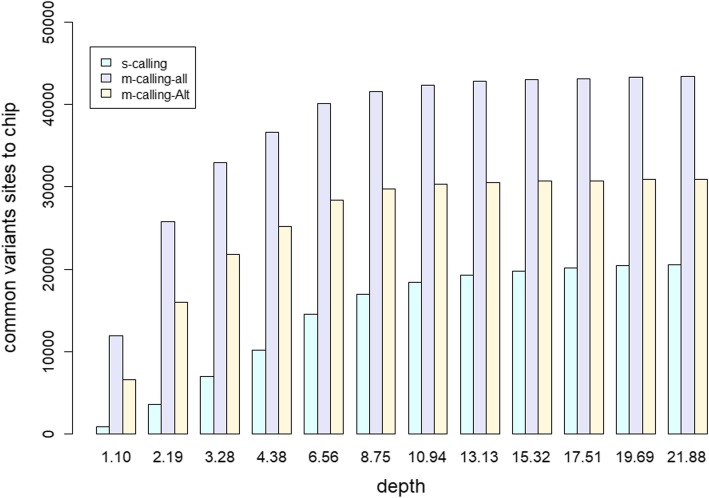
Common sites among Beadchip data. The common sites between each depth and beadchip dataset for single-sample calling (s-calling) and multisample calling (m-calling-all, m-calling-Alt). The number of common SNPs among the beadchip data (s-calling) was the average value for all three Yorkshire pigs for each depth. The multisample calling of all sites or only the sites with at least one nonreference allele is represented as m-calling-all and m-calling-Alt, respectively

**Fig. 7:**
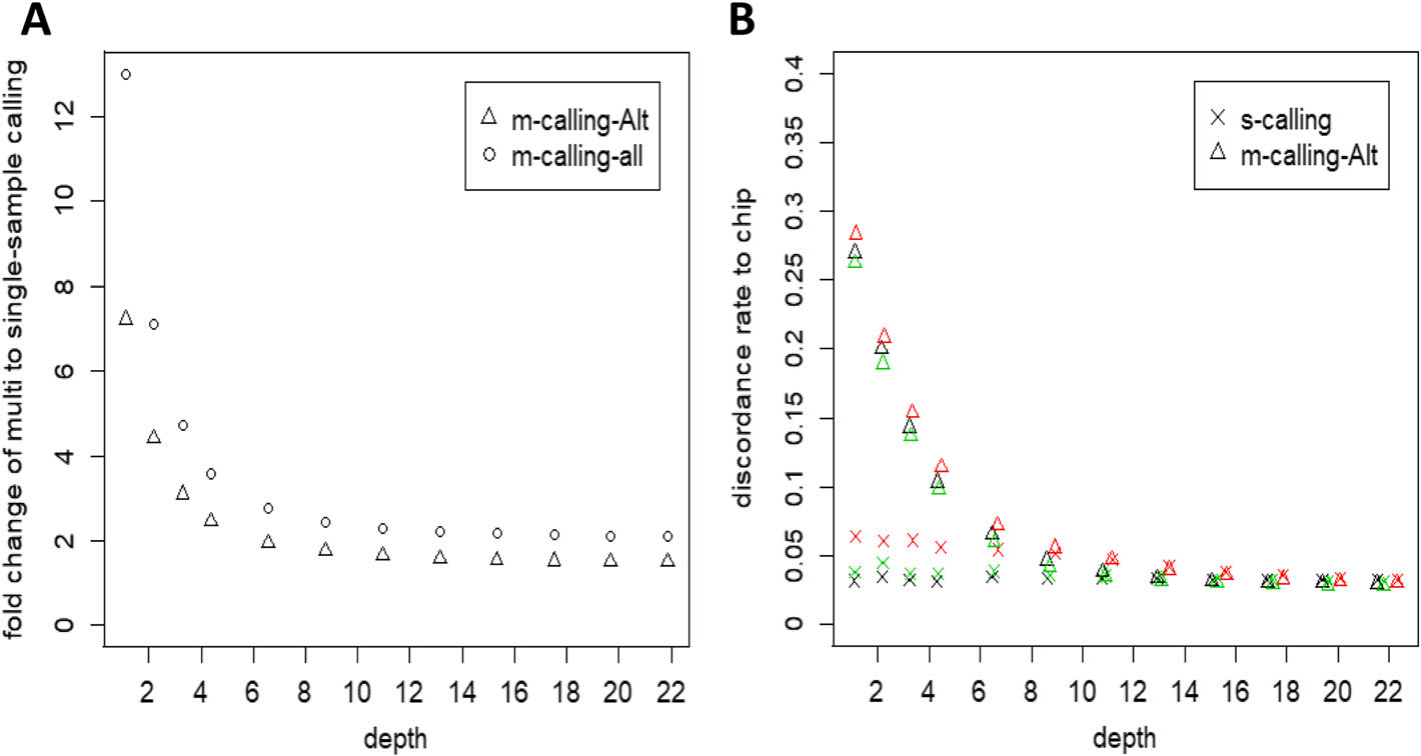
Fold change between multisample and single-sample calling and the quality of variants. (**a**) Fold change between multisample and single-sample calling. The ratio of common variants detected in multisample calling to that in single-sample calling. The statistics for multisample calling of all sites (m-calling-all) or only the sites with at least one nonreference allele (m-calling-Alt) are indicated separately. (**b**) Discordance rate with Beadchip data. Single-sample and multisample calling was compared to PorcineSNP80K BeadChip data for each Yorkshire subsample. For the comparison between single-sample calling and multisample calling, only the common sites of the samples between the PorcineSNP80K BeadChip and whole-genome sequencing SNPs that contained at least one nonreference allele were calculated

A total of 56,963 SNPs from the PorcineSNP80K BeadChip data remained after quality control. Figure 6 shows the common sites between each depth and beadchip dataset. These results showed that more common variants were discovered as the depth increased, and multisample calling revealed more variants than single-sample calling both for all variants and for only variants with at least one nonreference allele considered. The numbers of variants discovered via multisample calling were 2 times and 1.5 times greater than those obtained via single-sample calling at the greatest depth when all variant sites or only alternative reference sites were considered, respectively. The difference of the total variants discovered for multisample calling compared to single-sample calling increased as the sequencing depth decreased, as shown in Fig. 7a, especially when the depth was less than 4.38X.

Figure 7b shows the discordance rate with the SNP panel for Yorkshire under two scenarios of multisample and single-sample calling strategies. As expected, the discordance rate decreased with increasing depth. The two SNP calling strategies showed different performances. For single-sample calling, the discordance rate decreased slightly to 3.22% from 4.48% when the depth was increased from 1.10X to 21.88X, while multisample calling yielded a much higher discordance rate of 27.16% at a depth of 1.10X, which sharply decreased with increasing depth, then stabilized, with the discordance rate reaching 2.96% at a depth of 21.88X.

## Discussion

In this study, we aimed to provide a comprehensive understanding of the relationship between data quality and the quantity of SNP calling and genotyping in pig whole-genome sequencing. Three popular pig breeds, Duroc, Yorkshire and Landrace, were examined, and three boars from each breed were sequenced at approximately 20X depth. To the best of our knowledge, there have been no similar studies in other livestock, such as cattle or poultry. Our findings can therefore serve as a general guide for researchers to choose an optimal sequencing depth. We extracted paired read randomly from the original bam files at proportions of 0.05, 0.1, 0.15, 0.2, 0.3, 0.4, 0.5, 0.6, 0.7, 0.8 and 0.9 to mimic twelve different depths of genome sequencing data. Our results showed that the higher the depth of sequencing, the more novel variants were found, and the rate of false-positive variants was increased dramatically when the depth was lower than ~4X, especially when the depth was less than 2X. Additionally, the genome coverage of sequencing increased with depth, and a sequencing depth of 10X achieved 99% genome coverage (Fig. 4) and ensured high-quality genotyping for pigs (Fig. 5c; Fig. 5d; Fig. 7b). According to our findings, a depth of 10X was not only the point at which saturation was achieved for the function of coverage with depth (Fig. 4) but also the point at which the increase ratio for the total variants discovered slowed (Fig. 5b) and a plateau of discordance with the beadchip data was observed for multisample calling (Fig. 7b). More than 95% of the genome was covered at 4.35X, which is consistent with other investigations. Rashkin et al. [12] reported that 5-10X was a sufficient sequencing depth to detect common variants and identify associations for a fixed sequencing capacity in simulated data and human datasets. Although sequencing with greater depth can offer more information, many studies have indicated that low-coverage sequencing of large samples is much more cost-effective and powerful than the deep sequencing of fewer individuals. Li et al. [17] reported that when the frequency of variants is greater than 0.2%, only approximately 20% of the effort is needed to sequence 3000 individuals at 4X depth while achieving similar power to the sequencing of > 2000 individuals at 30X depth. Additionally, Keel’s research showed that sequencing a large number of individuals at 4-6X provides higher power than sequencing a smaller number of individuals at a great depth for rare variant detection [35]. The sequencing cost of the latter approach is much higher than that of the former. Therefore, considering the sequencing cost and convenience, a 4X depth is necessary to achieve more accurate genotyping of pigs since the number of false-positive variants increases dramatically when the depth is less than 4X. According to the related literature, 4X is also the depth used in the 1000 Genomes Project for the discovery of disease-associated variants associated with complex diseases in humans [36], and a 10X or greater depth has been used for the assessment of genome-wide genetic variation [37, 38] in a pig population or the detection of selection signatures [39, 40]. In general, 4X is an appropriate depth for genome-wide association studies, and 10X is an appropriate depth for accurate genotyping and population genetic studies.

To evaluate variant quality for each downsampled genome, the criteria of the novel rate, Ti/Tv ratio, and genotype concordance to the greatest depth (21.75X) were investigated. The novel rate gave us a general idea of the accuracy of variant calling and genotyping. The Ti/Tv ratio is an important criterion for assessing the quality of SNP calling [27], which is expected to be 2.1~2.2 for whole-genome variants [41]. Furthermore, a higher Ti/Tv ratio usually indicates higher accuracy of SNP calling [31, 41, 42]. Our results regarding the Ti/Tv ratio were in agreement with the expected ratio. Compared with Duroc and Landrace, Yorkshire presented a higher Ti/Tv. Low-coverage sequencing always introduces false-positive variants in NGS data analysis, but how low this coverage is remains unclear. Our results showed that the false-positive rate was increased significantly when the sequencing depth was less than ~4X (Fig. 4c; Fig. 5), while the novel rate was extremely high, and the Ti/Tv ratio and the concordance rate were also low, indicating that resequencing at depths lower than 4X could provide inaccurate variants. Moreover, the novel rate increased sharply when the depth was greater than 2.18X (Fig. 4c), which indicated that more false-positive variants were discovered when the depth was less than ~2X. With further decreases in sequencing depth, 2X is the lower boundary to ensure the quality and coverage of sequencing. This conclusion is in agreement with the simulation study by Fumagalli [23] showing that 2X is the minimum sequencing depth for obtaining accurate estimates of allele frequencies and identifying polymorphic sites. For the comparison of SNP calling across the three pig breeds, the coverage and concordance with the greatest depth as a function of depth showed no difference across breeds; however, the novel rate and Ti/Tv ratio as a function of depth differed between Yorkshire and the other two breeds (Duroc and Landrace). According to B. N. Keel et al. [41], the average Ti/Tv ratios were 2.183, 2.206 and 2.243 for 12 Duroc, 12 Landrace and 48 Yorkshire-Landrace composite sows based on Illumina HiSeq 2500 technology and alignment to the Sscrofa10.2 reference genome, resulting in a mean of 6.1-fold coverage per genome. In addition, the Ti/Tv ratio varies greatly by genome region and function [31]; the Ti/Tv ratio is generally approximately 3.0 for exome sequencing data and approximately 2.0 outside of exome regions [43]. To our knowledge, it is most likely that biological factors lead to the differences in the Ti/Tv ratio across breeds.

Genotype imputation is widely used in whole-genome association studies and genomic prediction/selection, where a number of individuals are sequenced at a great depth as a reference panel, after which the imputation of individuals on the basis of SNP chip data or low-depth sequencing would be cost efficient. Sequencing data are becoming increasingly important for purposes such as association studies, genomic selection, etc., in which large samples are essential. Thus, to balance sequencing cost and efficiency, the sequencing strategy should be taken into account in practice. Two-stage sequencing has been suggested as a strategy in which some portion of a sample is first sequenced at high coverage as a reference panel, after which the larger sample is sequenced at low coverage, which has proved to be powerful, effective and practical approach [18]. Moreover, STITCH [44], which is a method that was developed for the imputation of genotypes based on sequencing data without the use of additional reference panel or array data, achieves a high imputation accuracy for ultralow-coverage sequencing. The approach resulted in accuracy values of 0.948 and 0.922 for sequencing data for outbred mice (0.15X) and Han Chinese people (1.7X), respectively. Furthermore, GeneImp [45] was developed for the imputation of ultralow-coverage sequencing data (<1X) with a reference panel, which achieved an even higher accuracy of 0.9. With the development of algorithms and software for low-coverage sequencing or even ultralow-coverage sequencing, additional applications of low-coverage sequencing may be developed, and our research can provide basic guidance for such applications.

In this study, we also compared single-sample calling and multisample calling algorithms. The single-sample calling algorithms were simple, making use solely of reads collected at a single genome position for that sample. However, the multisample calling algorithm included all sample information for a single site. According to our results, multisample calling revealed more variants than single-sample calling, and the lower depth of sequencing, the greater the difference was, with 13-fold and two-fold differences in the numbers of variants discovered via multisample calling compared to single-sample calling when sequencing was performed at 1X and 22X, respectively. Additionally, multisample calling produced more false-positive variants than single-sample calling when the depth was less than 10X. Similar results were found in Bizon’s research [46] and Liu’s research [47] conducted in a Native American population and another human dataset, respectively. Our results further confirmed the marginally lower nonreference discrepancy value observed for identified single-sample variants than variants obtained via the multi-sample method in sequence data from 65 cattle [48]. Our results suggested that stricter quality control parameters should be implemented in multisample calling, especially when the depth is less than 10X.

## Conclusion

In this study, we explored the relationship between sequencing depth and whole-genome coverage, discovery power, and the accuracy of SNP calling across three pig breeds, Duroc, Landrace and Yorkshire. The genotyping accuracy of the sequencing data was validated with PorcineSNP80K BeadChip data for Yorkshire pigs as well. In addition, multisample and single-sample strategies for SNP calling were compared. Our results showed that a depth of 10X was the point at which saturation was reached for the function of coverage, covering 99% of the whole pig genome, accounting for 84.42% of the variants obtained from the deepest genome coverage (21.75X in this study), ensuring good quality of variants from the aspects of the novel rate, Ti/Tv ratio, and beadchip validation. Additionally, more false-positive variants were detected when the depth was less than 4X, suggesting that 4X is the low boundary for reasonable sequencing quality. Compared to single-sample calling, multisample calling was more sensitive, especially at lower depths, and more false-positive variants were detected as well; stricter quality control parameters should be implemented in multisample calling.

## Data Availability

The whole-genome sequencing data for Yorkshire boars obtained in the current study are available from the corresponding author upon reasonable request. The datasets for Duroc and Landrace pigs are available in the NCBI Sequence Read Archive under accession PRJNA343658.

## Abbreviations

GATK: The Genome Analysis Toolkit
LoF: Loss-of-function
NGS: Next Generation Sequencing
ORF: Open Reading Frame
SNP: Single Nucleotide Polymorphism
Ti/Tv ratio: Transition/Transversion ratio
